# Simultaneous Analysis of Cyanotoxins β-N-methylamino-L-alanine (BMAA) and Microcystins-RR, -LR, and -YR Using Liquid Chromatography–Tandem Mass Spectrometry (LC-MS/MS)

**DOI:** 10.3390/molecules28186733

**Published:** 2023-09-21

**Authors:** Sercan Pravadali-Cekic, Aleksandar Vojvodic, Jake P. Violi, Simon M. Mitrovic, Kenneth J. Rodgers, David P. Bishop

**Affiliations:** 1 Hyphenated Mass Spectrometry Laboratory (HyMaS), University of Technology Sydney, Sydney, NSW 2007, Australia; sercan.pravadali-cekic@uts.edu.au (S.P.-C.);; 2 School of Chemistry, University of New South Wales, Sydney, NSW 2033, Australia; j.violi@unsw.edu.au; 3 School of Life Sciences, University of Technology Sydney, Sydney, NSW 2007, Australia; simon.mitrovic@uts.edu.au (S.M.M.); kenneth.rodgers@uts.edu.au (K.J.R.)

**Keywords:** BMAA, microcystins, cyanotoxins, cyanobacteria, method validation

## Abstract

β-N-methylamino-L-alanine (BMAA) and its isomers, 2,4-diaminobutyric acid (2,4-DAB) and N-(2-aminoethyl)-glycine (AEG), along with microcystins (MCs)-RR, -LR, and -YR (the major MC congeners), are cyanotoxins that can cause detrimental health and environmental impacts during toxic blooms. Currently, there are no reverse-phase (RP) LC-MS/MS methods for the simultaneous detection and quantification of BMAA, its isomers, and the major MCs in a single analysis; therefore, multiple analyses are required to assess the toxic load of a sample. Here, we present a newly developed and validated method for the detection and quantification of BMAA, 2,4-DAB, AEG, MC-LR, MC-RR, and MC-YR using RP LC-MS/MS. Method validation was performed, assessing linearity (r^2^ > 0.996), accuracy (>90% recovery for spiked samples), precision (7% relative standard deviation), and limits of detection (LODs) and quantification (LOQs) (ranging from 0.13 to 1.38 ng mL^−1^). The application of this combined cyanotoxin analysis on a culture of *Microcystis aeruginosa* resulted in the simultaneous detection of 2,4-DAB (0.249 ng mg^−1^ dry weight (DW)) and MC-YR (4828 ng mg^−1^ DW). This study provides a unified method for the quantitative analysis of BMAA, its isomers, and three MC congeners in natural environmental samples.

## 1. Introduction

Cyanobacteria are oxygenic, photosynthetic, prokaryotic microorganisms that are known to inhabit terrestrial, marine, and freshwater environments. Under optimal environmental conditions, cyanobacteria proliferate rapidly and form ‘algal’ blooms. Cyanobacterial blooms have a number of ecological and epidemiological impacts [1], including the death of aquatic organisms, decline in biodiversity, disruption of fresh water supplies, and the production of allergens and toxic metabolites known as cyanotoxins [1,2,3]. The impact of cyanobacterial blooms extends to fatalities of wildlife, livestock, and pets, as well as issues related to human health. There are a range of cyanotoxins that are routinely monitored, such as the hepatotoxic microcystins (MCs), and there are also a number of toxins that are not yet well understood, such as the neurotoxin β-N-methylamino-L-alanine (BMAA), which is rapidly gaining attention for its association with neurodegenerative diseases [4,5,6].

**Figure 1:**
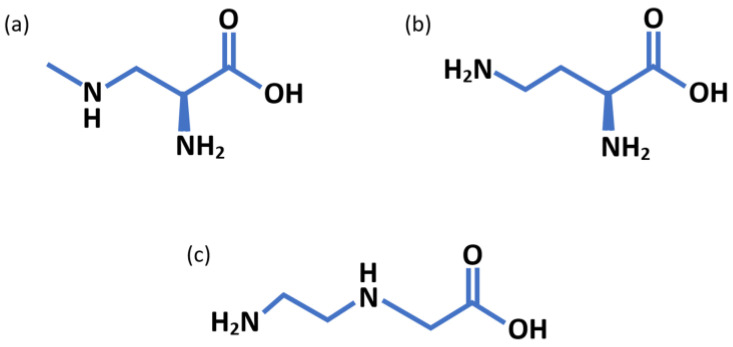
BMAA and isomer chemical structures: (**a**) β-N-methylamino-L-alanine (BMAA), (**b**) L-2,4-diaminobutyric acid (2,4-DAB), and (**c**) N-(2-aminoethyl)glycine (AEG).

BMAA is a non-protein amino acid (NPAA) produced by a wide range of cyanobacteria from various aquatic ecosystems, with up to 90% of cyanobacteria and some diatoms having been found to produce BMAA [3,7]. It has been proposed that BMAA is associated with the onset of amyotrophic lateral sclerosis/parkinsonism dementia complex (ALS/PDC) [8], a complex neurological disorder observed in areas with potentially high BMAA exposure such as the Mariana Islands (Guam and Rota), Kii Peninsula (Japan), and southeastern Irian Jaya [9]. Surveys in the 1950s reported that ALS on Guam was 50 to 100 times more prevalent than in the USA and other Western countries [9]. BMAA has two main constitutional isomers, L-2,4-diaminobutyric acid (2,4-DAB) and N-(2-aminoethyl)glycine (AEG) (Figure 1), which are commonly found together; 2,4-DAB, like BMAA, is also considered to be neurotoxic, with similar excitotoxic properties, and is widespread in nature [10]. A recent in vitro study showed that the simultaneous exposure of human neuroblastoma cells to 2,4-DAB and BMAA decreased cell viability more than exposure to BMAA or 2,4-DAB alone, suggesting that the presence of 2,4-DAB increases the toxicity of BMAA [11]. This study also showed that AEG was the least toxic of the three isomers, with the exposure of human neuroblastoma cells (SH-SY5Y) to AEG being up to four times less toxic than exposure to 2,4-DAB or BMAA [11]. In another study, AEG was found to be up to ten thousand times less toxic than BMAA in an *Artemia salina* bioassay [12]. In contrast, AEG was reported to be more toxic than BMAA and 2,4-DAB in mixed cortical cell cultures from foetal mice [13].

**Figure 2:**
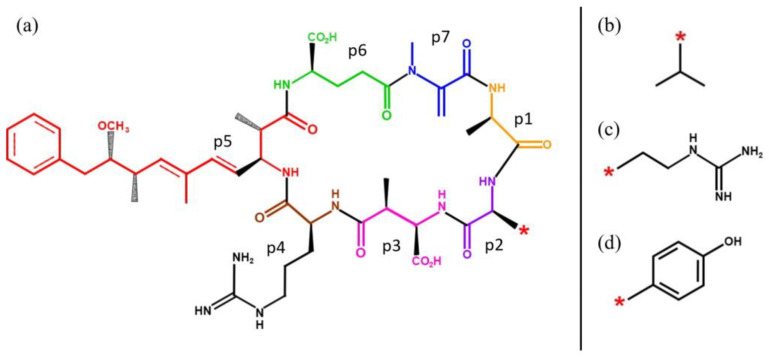
Chemical structures of MC-LR, MC-RR, and MC-YR: Chemical structure for (**a**) MC-*R, (**b**) MC-LR, (**c**) MC-RR, and (**d**) MC-YR.

MCs are cyclic heptapeptides produced by various genera of freshwater cyanobacteria worldwide. MCs were first isolated from the cyanobacterium *Microcystis aeruginosa* [14]. They have a general cyclic structure containing five non-protein amino acids and two variable L-amino acid functional groups. From the array of MCs, MC nomenclature is indicative of the amino acid functional groups and their positions within the MC chemical structure [15]. There are 279 characterised congeners that have been reported in the literature [15,16,17], and these are the most widespread cyanotoxins, with genetics and environmental factors contributing to their structural diversity [16,18]. The most common detected congeners of MCs are MC-LR, MC-RR, and MC-YR (Figure 2), containing the amino acids leucine (L), arginine (R), and tyrosine (Y) [19,20,21,22]. MC-LR is also the most toxic, where a provisional guideline value of 1.0 ng mL^−1^ has been set by the WHO to ensure the safety of drinking water [23]. 

MCs are highly potent hepatotoxins, high doses of which can cause severe acute outcomes and even death as a result of liver damage. Acute doses lead to liver necrosis, intrahepatic haemorrhage, and shock, whereas lower MC doses have been shown to lead to a slower onset of liver and kidney failure. MC exposure can also lead to mitochondrial alterations, affect intracellular calcium levels, and cause oxidative stress, all of which contribute to hepatotoxicity [24]. Since MCs pose severe health risks from both acute and chronic exposure, including hepatotoxicity [25] and nephrotoxicity [26], as well as cardiovascular toxicity [27] and even reproductive toxicity [28], it is imperative to ensure that human exposure is minimised; as such, their presence is monitored and regulated in a number of countries, including Australia [29], the USA [30], Norway [31], and many European countries [32,33]. 

Water quality assessments for the presence of cyanotoxins can be problematic due to the complex nature of cyanobacterial blooms, which contain multiple species of cyanobacteria and several classes of cyanotoxins, including MCs and BMAA, AEG, and 2,4-DAB. A recent increase in awareness of the hazards presented by cyanobacterial toxins has resulted in the development of various methods, from biological-based screening methods to more sophisticated and confirmatory analytical techniques [34]. Currently, some regulatory authorities issue warnings to the public when cyanobacterial blooms of species that are known to produce MCs occur, based on total cell numbers [35]. However, an understanding of the toxin concentrations in water samples is more useful to elucidate the risks to water users and is covered in some guidelines, such as those of the WHO [23]. 

Liquid chromatography–tandem mass spectrometry (LC-MS/MS) is currently the most common analytical technique for the separation, detection, and quantification of BMAA and its isomers, AEG and 2,4-DAB [36]. For the analysis of BMAA, AEG and 2,4-DAB pre-column derivatisation techniques such as 6-aminoquinoly-N-hydroxysuccinimidyl carbamate (AQC) [37], 9-fluorenylmethyl chloroformate (FMOC) [38], and ethyl- or propyl-chloroformate (ECF/PCF) [39] are generally employed to increase molecular mass to allow for reverse-phase separation and increase ionisation efficiency [40]. More recently, LC-MS has also been the preferred method for the identification and quantification of multiple MC congeners, due to its ability to easily fragment and analyse native MCs whilst maintaining good retention on reverse-phase columns [41,42,43,44].

The analysis of cyanotoxins has typically focused on only one class of toxin in a single sample, and while several publications exist for multiclass cyanotoxin analysis of various sample matrices, there have been few studies outlining methods that include the simultaneous screening and quantification of both MCs and BMAA within a single biological sample—more specifically, algal samples [45,46,47,48,49,50]. It is important to employ multiclass cyanotoxin analyses to fully understand the toxic load of cyanobacterial blooms and to minimise the risk of human and animal exposure. Studies on combined cyanotoxin analysis have included BMAA and anatoxin [46]; MCs and cylindrospermopsin (CYN) [47,48]; a combination of MCs, nodularin (NOD), anatoxins, CYN, and saxitoxins [45]; and an extensive combination of 12 different MC congeners, CYN, ANA, and NOD, including two other algal toxins: okadaic and domoic acids [49]. Recently, a combined multiclass cyanotoxin analysis method was developed for the extraction, preconcentration, and determination of eight cyanotoxins, including MC-LR, MC-RR, nodularin, cylindrospermopsin, anatoxin-a, BMAA, 2,4-DAB, and AEG, using solid-phase extraction (SPE) sample treatment in tandem with hydrophilic interaction liquid chromatography (HILIC)-MS/MS [50]. Another study analysed seven of the aforementioned cyanotoxins, excluding cylindrospermopsin, on spirulina-based supplements through a newly developed solid–liquid extraction method combined with SPE and analysed by HILIC-MS/MS [51]. However, HILIC is known to be temperamental, with poor peak shape, separation efficiency, and reproducibility. It is also susceptible to decreased sensitivity due to ion suppression as a consequence of the high buffer concentrations that are generally utilised, which may therefore require a compromise between sufficient ion intensity and ideal chromatography [52]. Therefore, we developed and validated a sensitive and efficient RPLC-MS/MS method for the simultaneous detection and quantification of BMAA, AEG, 2,4-DAB, MC-LR, MC-YR, and MC-RR. 

## 2. Results and Discussion

### 2.1. Method Development 

#### 2.1.1. MC Protonation

The molecular weight of MC-RR is 1038.2 g mol^−1^, and it therefore produces a singly charged *m*/*z* of 1039.2 [M+H]^+^ in the electrospray ionisation source. However, the two arginine (R) residues of MC-RR both become protonated in the process of ionisation, resulting in a double-protonated molecule at 520 *m*/*z* [M+2H]^2+^ [53]. Generally, the electrospray ionisation process of MC-LR and MC-YR results in singly charged ions [M+H]^+^ [54], although considering the double-protonation behaviour of MC-RR during ionisation, double-protonation of MC-LR and -YR was also investigated. There is strong evidence suggesting that MCs containing the -R residue may have an additional protonation site, either on amide nitrogens in cyclic peptide bonds or on the Adda moiety of MCs [16,55]. Various studies have used the double-protonated MRM transitions for MC-LR and MC-YR (MC-LR, 498 → 135 *m*/*z* and MC-YR, 523 → 135 *m*/*z*) to determine the concentrations of MCs using LC-MS/MS, with improved LODs [56,57,58]. The inclusion of the double-protonated transitions in this method also increased the peak intensities for MC-LR and MC-YR, consistent with the literature [45].

#### 2.1.2. Derivatisation Considerations

For RPLC-MS/MS, derivatisation is essential for the detection of BMAA, AEG, and 2,4-DAB to improve chromatographic separation and sensitivity. The derivatisation method of choice for the simultaneous analysis of BMAA, AEG, 2,4-DAB, and the MCs was AQC. A critical point to consider in the combination of these cyanotoxins is whether derivatisation, specifically AQC, affects the MCs. A simple experiment was conducted in which the MCs were analysed under three conditions: (1) 10 µL of 1 ppm MCs + 90 µL of MeOH (control); (2) 10 µL of 1 ppm MCs + 90 µL of MeOH, incubated for 10 min at 55 °C; and (3) 10 µL of 1 ppm MCs + 20 µL of derivatising reagent + 70 µL of borate buffer, incubated for 10 min at 55 °C. The peak area comparison results indicated that the MCs were not derivatised or affected by the derivatisation process. The likelihood of the AQC tag reacting with the primary or secondary amines of amino acid residues in MCs is low, as it is generally more difficult to derivatise large molecules due to their significantly slower chemical reaction rates. Derivatisation of large molecules is not impossible; however, the efficiency of larger molecules’ derivatisation is significantly lower than that of smaller, more reactive molecules [59]. Furthermore, the energy of activation required to derivatise a primary amino group in a large molecule like MC is much greater than the activation energies required to derivatise small molecules like BMAA [59].

#### 2.1.3. Chromatographic Optimisation

**Figure 3:**
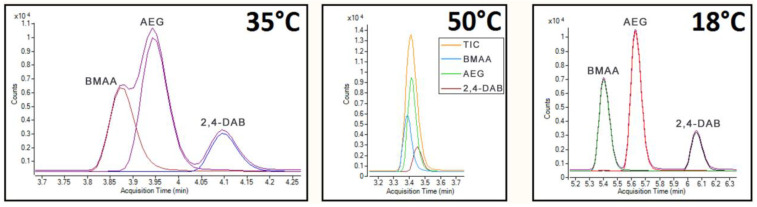
Total ion count (TIC) and MRM chromatograms showing the variable effect of column oven temperature on BMAA, AEG, and DAB peak separation.

In determining the chromatographic conditions for the combined cyanotoxin analysis, each class of cyanotoxin compounds was tested under various chromatographic conditions, with acetonitrile (+0.1% formic acid) as the organic mobile phase. It was initially observed that 10% organic mobile phase in isocratic mode did not provide an acceptable resolution between BMAA, AEG, and 2,4-DAB within a reasonable chromatographic timeframe. Previous studies have used gradient elution for the separation of AQC-derivatised BMAA, AEG, and 2,4-DAB [60,61]; thus, gradient elution was also explored to further develop the method. Chromatographic conditions alone (i.e., elution mode, mobile phase composition, and column) did not produce a sufficient separation resolution between BMAA, AEG, and 2,4-DAB. To achieve adequate separation of BMAA, AEG, and 2,4-DAB, various column temperatures were explored. Column temperature assists in chromatographic separation by directly impacting the analyte exchange rates between the mobile and stationary phases; the higher the temperature, the faster the exchange rate and, thus, the shorter the retention time. However, increasing the column temperature reduced the resolution between BMAA, AEG, and 2,4-DAB, while lowering the column temperature provided better separation performance and, thus, better resolution, albeit with slightly longer retention times. Figure 3 shows the effect of column temperature on the separation of BMAA, AEG, and 2,4-DAB. The optimal column temperature for the separation of BMAA and its isomers was determined to be 18 °C, providing a resolution factor of 1.65 between BMAA and AEG and 2.68 between AEG and 2,4-DAB.

**Figure 4:**
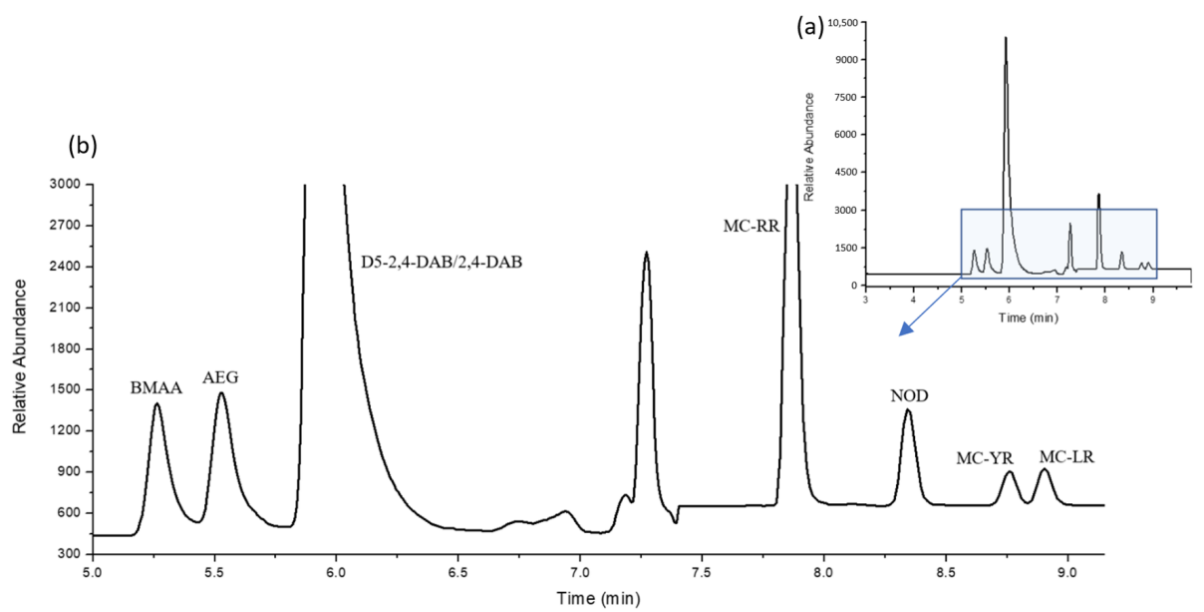
TIC representation of the separation of 10 ng mL^−1^ combined standard of cyanotoxin NPAAs (BMAA, AEG, 2,4-DAB, and D5-2,4-DAB) and cyclic peptides (nodularin, MC-RR, MC-YR, and MC-LR) under optimal chromatographic conditions: (**a**) entire chromatographic range (3.0–9.8 min); (**b**) zoomed chromatographic window (5.0–9.5 min).

MCs have higher molecular weights compared to BMAA and required stronger mobile-phase conditions for timely elution, with chromatographic conditions of 33% to 45% acetonitrile (+0.1% *v*/*v* formic acid) separating nodularin, MC-YR, MC-LR, and MC-RR. Good separation between the three MC congeners was achieved with the reduced column temperature (18 °C) required for separating BMAA, AEG, and 2,4-DAB. The chromatographic information obtained from both cyanotoxin classes was then combined, resulting in a single method that allowed for the adequate separation of each analyte, the final analytical conditions of which are detailed in the Section 3. Figure 4 illustrates the chromatographic separation of a 10 ng mL^−1^ standard containing all of the cyanotoxins in a single run using this newly developed multiclass analytical method.

### 2.2. Method Validation

Recently, an SPE-HILIC-MS/MS method was published for the extraction, preconcentration, and analysis of multiclass cyanotoxins, including MC-LR, MC-RR, nodularin, cylindrospermopsin, anatoxin-a, BMAA, AEG, and 2,4-DAB [50]. The method involved two different SPE cartridges (Strata-X and Oasis MCX) in tandem to allow for the extraction and preconcentration of all of the classes of cyanotoxins in reservoir waters, which were then analysed using a zwitterionic HILIC column (LC-MS/MS). The method validation involved a procedural calibration, which underwent the SPE preconcentration step prior to LC-MS/MS analysis, assisting in achieving low LODs ranging from 0.001 to 0.015 ng mL^−1^, LOQs ranging from 0.004 to 0.05 ng mL^−1^, and maximum precision of 14.1% for the same classes of cyanotoxins. The recovery rates were based on several spiked reservoir water samples, with 70–100% (%RSD < 17.5%) for most classes of cyanotoxins, except for BMAA and DAB, which showed a reduction in recovery rates in reservoir waters with high cation contents. Although the authors achieved the development of a method for multiclass cyanotoxins, including MC-RR, MC-LR, BMAA, AEG, and 2,4-DAB, their study was limited to water samples, and its application to cyanobacteria matrices (along with the limitations of HILIC, as previously mentioned) is not feasible. 

HILIC is a technique that is well suited to the analysis of small molecules; however, its application to the analysis of BMAA and its isomers has shown poor performance across all validation factors, as demonstrated by Tymm et al. [62]. Analyses using HILIC generally fail to detect BMAA in cyanobacteria more often than RPLC techniques [63]. In addition, RPLC methods are more robust and do not require lengthy re-equilibration procedures for sensitive analyses [62]. Thus, the application of the AQC-derivatised RP LC-MS/MS method was deemed most appropriate for the analysis of BMAA, AEG, 2,4-DAB, and MCs-RR, -LR, and –YR, as well as for biological samples like cyanobacteria, providing fast and reliable results. 

It is important to note that another isomer of BMAA, β-amino-N-methyl-alanine (BAMA), may be present; however, it is not as widely studied as the other BMAA isomers and has limited ecotoxicological data [38]. BAMA is known to produce the same fragmentation patterns as BMAA in MS/MS when derivatised with AQC [64,65]. Therefore, insufficient chromatographic separation may result in the false-positive identification and quantification of BMAA. Some studies suggest that the detection of BMAA in the literature is misidentified and overestimated due to the possible interference of the BAMA isomer [66,67]. However, the inclusion of ion ratio comparisons can be used to distinguish BMAA from BAMA and other interfering analytes, as a study showed that BMAA and BAMA have different ion ratios between 119.08 and 258.09 mass-to-charge (*m*/*z*), with ratios of 4.4 and 18.4 for BMAA and BAMA, respectively [64]. Therefore, any shift in ion ratio between 119.08/258.09 *m*/*z* is indicative of compromised BMAA identification, and this was not observed here.

The proposed LC-MS/MS method was validated in terms of linearity, limits of detection (LODs), limits of quantification (LOQs), accuracy, and intra- and inter-day precision. The linearity and calibration range for each analyte were examined with 10-point internal standard calibration curves, ranging from 0.5 to 500 ng mL^−1^, with each calibration point run in five replicates. The correlation coefficients (r) were greater than 0.996 for all target analytes. The linear range for BMAA, AEG, and 2,4-DAB was 2–500 ng mL^−1^, and the linear range of the MC compounds was 0.5–500 ng mL^−1^, resulting in lower LODs and LOQs than for BMAA and its isomers, AEG and 2,4-DAB, within the WHO guidelines with respect to MCs. These results are presented in Table 1. The accuracy of the method was determined using a spike-recovery test, whereby a sample matrix was spiked with 10 ng mL^−1^ of each analyte and compared to the 10 ng mL^−1^ calibration standard. The recovery rates are summarised in Table 1. All analytes produced good recovery rates, ranging from 85.4% (2,4-DAB) to 109.3% (MC-LR). The method’s precision was evaluated in terms of inter- and intra-day repeatability of peak areas and retention times at the concentration of 10 ng mL^−1^, and it was reported as the % relative standard deviation (%RSD) (Table 2). The repeatability tests produced acceptable levels of %RSD for the peak area, with the highest %RSD being 6.6% for AEG during inter-day analysis. The linearity data obtained from this method validation, alongside the recovery rates and repeatability results, suggest that this method is adequate for sample analysis.

### 2.3. Cyanotoxin Identification in Cyanobacterial Samples 

**Figure 5:**
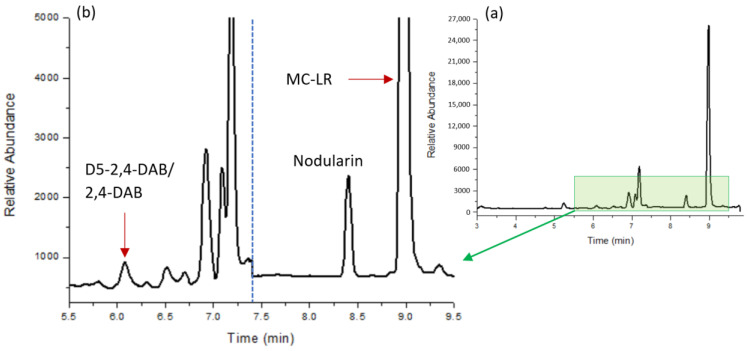
Chromatographic representation of an MA-cultured sample showing positive 2,4-DAB and MC-LR detection using the combined cyanotoxin LC-MS method: (**a**) entire chromatographic range (3.0–9.8 min); (**b**) zoomed chromatographic window (5.5–9.5 min) chromatogram showing 2,4-DAB and MC-LR peaks.

A cyanobacterial bloom scum sample (a mixture of *Microcystis flos aquae* and *Dolichospermum crassum*) and a cyanobacterial culture of MA were tested for BMAA, AEG, 2,4-DAB, MC-RR, MC-LR, and MC-YR. The samples underwent BMAA, AEG, 2,4-DAB, and MC extraction as per the protocol outlined in the Materials and Methods. Table 3 lists the average dry weight (DW) concentrations of each cyanotoxin detected. MA was found to contain the 2,4-DAB isomer (0.249 ng mg^−1^ DW) and MC-YR (4828 ng mg^−1^ DW) (see Figure 5). The environmental scum sample collected from Gunbower, Victoria contained MC-RR and MC-LR (134.9 and 41.17 ng mg^−1^, respectively), with no BMAA or its isomers detected. 

Cyanobacterial blooms may contain a variety of cyanobacteria species, resulting in the potential for the co-occurrence of multiclass cyanotoxins, such as MCs, anatoxins, and NPAAs, with two-, three-, and up to four-class co-occurrence [68]. Due to anthropogenic factors, the occurrence, intensity, and duration of cyanobacterial blooms are increasing, posing a greater risk to human health and ecosystems, and as the possible routes of exposure increase—such as through the contamination of drinking water and crops—the potential risks to human health are magnified. Algae-based food supplements have also been on the rise, with studies showing their positive health effects through human consumption [69,70,71]. However, the regulation of the promotion and consumption of algae is not strictly controlled, where in Europe up to 150 algae species have been identified in regular consumption, with only one-fifth of them having been approved by the EU Novel Food legislation [72]. The MCs are well-researched and regularly monitored cyanotoxins that are known to be produced by common taxa such as *Microcystis*, *Dolichospermum*, and *Planktothrix* [68]. BMAA has also been detected in these cyanobacteria genera [73]. The MA sample that was analysed in this study illustrated the co-occurrence of multiclass cyanotoxins produced by a single cyanobacterial species, with the detection of 2,4-DAB and MC-LR. 

Furthermore, with the recent discovery of the combined toxic effects of BMAA and 2,4-DAB on human neuroblastoma cells in vitro, it is important to detect and assess the presence of both BMAA and 2,4-DAB, along with their quantities, as the cytotoxic effects are greater than those of BMAA or 2,4-DAB alone [11]. The co-occurrence of multiple cyanotoxins may exacerbate potential health risks. As our knowledge of neurotoxins such as BMAA increases, there is a need for it to be included in multiclass cyanotoxin analysis methods to better facilitate routine monitoring and testing of drinking water, foods, supplements, and water bodies. 

There have been several studies looking at a range of cyanotoxin classes for various sample matrices [49,74]. However, only recently have methods included both the MC and BMAA classes within the range of analysed cyanotoxins, albeit these studies have been limited to water samples [75] and HILIC-MS methods with the inclusion of SPE preparation techniques [50]. The most widespread cyanotoxins are MCs, with MC-LR, MC-RR, and MC-YR being the most commonly detected congeners [19,20] and MC-LR being the most toxic variant [76]. Governments that regulate the use of waterways affected by cyanobacterial blooms do so based on the presence of species that produce these congeners; therefore, they were selected in the development of this multiclass method. The inclusion of more MC congeners would provide additional information on the potential toxicity of a bloom [19]; however, potential use of this method by contract laboratories performing routine monitoring to meet regulatory requirements would be exceeded. BMAA and its isomers were selected due to the increase in knowledge of their toxicity and links to neurodegenerative diseases. There is still conjecture as to the mechanisms of BMAA’s and 2,4-DAB’s toxicity, and they are not currently monitored by regulatory bodies; however, once these are established, it may be necessary to include them in routine analysis methods.

## 3. Materials and Methods

### 3.1. Chemicals and Reagents

#### 3.1.1. Standards

Standards of L- β-N-methylamino-L-alanine hydrochloride (≥97%), HPLC-grade MC standard mix (-RR, -YR, and -LR), and nodularin methanol solution were purchased from Sigma-Aldrich (Castle Hill, NSW, Australia). L-2,4-diaminobutyric acid dihydrochloride (≥95%) and N-(2-aminoethyl)-glycine (≥97%) were purchased from Toronto Research Chemicals Inc. (North York, ON, Canada). D-2,4-diaminobutyric-2,3,3,4,4-d5 acid dihydrochloride was purchased from CDN Isotopes (Pointe-Claire, QC, Canada). BMAA, AEG, and 2,4-DAB were derivatised using the AccQ-Tag Ultra Derivatization Kit, supplied by Waters Corporation (Milford, MA, USA).

#### 3.1.2. Samples

A cyanobacterial scum was collected from off-take (‘diverted’) water from a weir in Gunbower, Victoria, Australia (collection date: 9 September 2016). A cultured cyanobacteria strain of *Microcystis aeruginosa* was also collected from the Faculty of Science, UTS Australia. The samples were freeze-dried at 0.1 mbar and −80 °C prior to extraction. 

### 3.2. Sample Extraction

#### 3.2.1. Extraction of BMAA, AEG, and 2,4-DAB 

**Figure 6:**
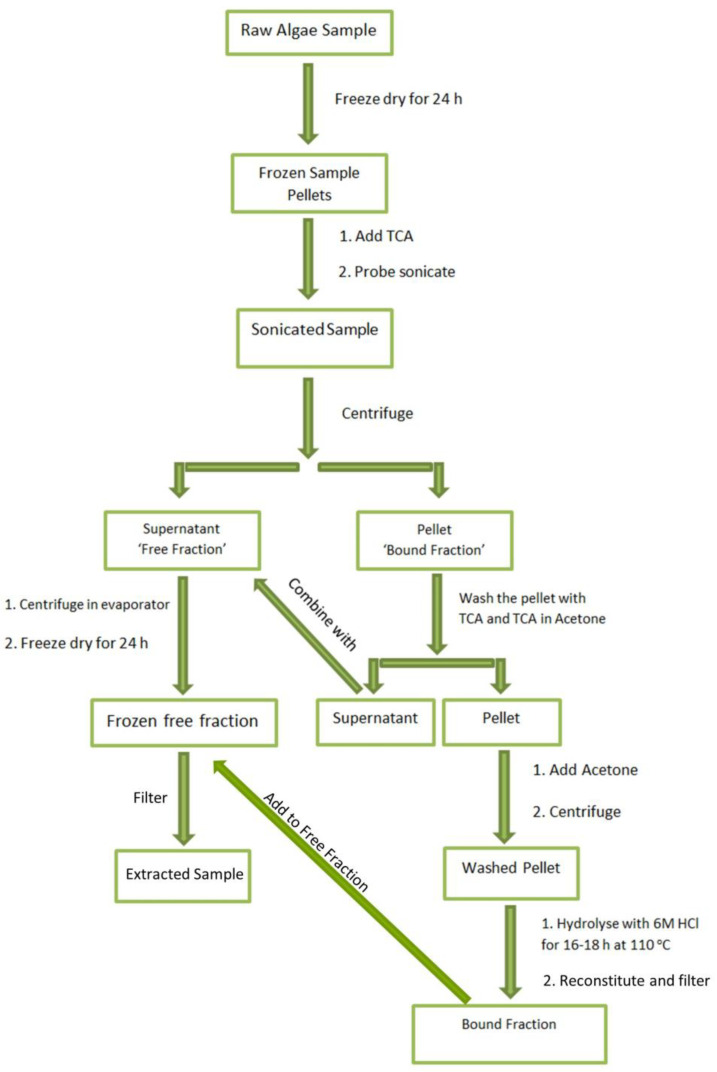
Schematic of the BMAA, AEG, and 2,4-DAB sample extraction workflow process.

The extraction of BMAA, AEG, and 2,4-DAB was carried out based on an existing and validated method [77,78]. Approximately 15 mg of dry cyanobacterial mass was lysed twice in 300 µL of 10% *w*/*v* trichloroacetic acid (TCA) via probe sonication for 1 min at 70% amplitude, followed by overnight precipitation at 4 °C. The precipitated pellet was centrifuged at 3000× *g* and 4 °C for 15 min, and the supernatant was transferred into a ‘free fraction’ tube. Another 300 µL of 10% *w*/*v* TCA was added to the pellet, and the centrifuge and supernatant transfer step was repeated. This step was repeated for the third time with the use of 10% TCA/acetone instead of 10% TCA. The remaining pellet (‘bound fraction’) was transferred to an engraved shell vial with 100% acetone. The shell vials containing the pellets were centrifuged, and the supernatant was transferred into the ‘free fraction’ tube. The ‘free fraction’ tube was then placed into a SpeedVac concentrator for 30 min, frozen in a −80 °C freezer, and freeze-dried at 0.1 mbar and −80 °C. The shell vials that contained the pellets were dried using the SpeedVac concentrator and prepared for acid hydrolysis by adding 1 mL of 6 M HCl to the bottom of the hydrolysis vial from an Eldex Hydrolysis/Derivatization Workstation (Eldex Laboratories, Inc. Napa, CA, USA), followed by the insertion of the shell vials. The oxygen was removed from the hydrolysis vial by subjecting it to a vacuum pump, lowering the pressure to 300 mbar, and filling it with nitrogen gas for three cycles. The sample then underwent hydrolysis overnight in an oven set at 110 °C. The hydrolysed pellet, or ‘bound fraction’, was reconstituted with 200 µL of 500 ng mL^−1^ D5-DAB internal standard and then transferred to the corresponding ‘free fraction’. The samples were then filtered through a 0.2 μm pore membrane filter under centrifugation, resulting in the ‘Final Product’. Figure 6 schematically describes the BMAA, AEG, and 2,4-DAB extraction process.

#### 3.2.2. MC Extraction

**Figure 7:**
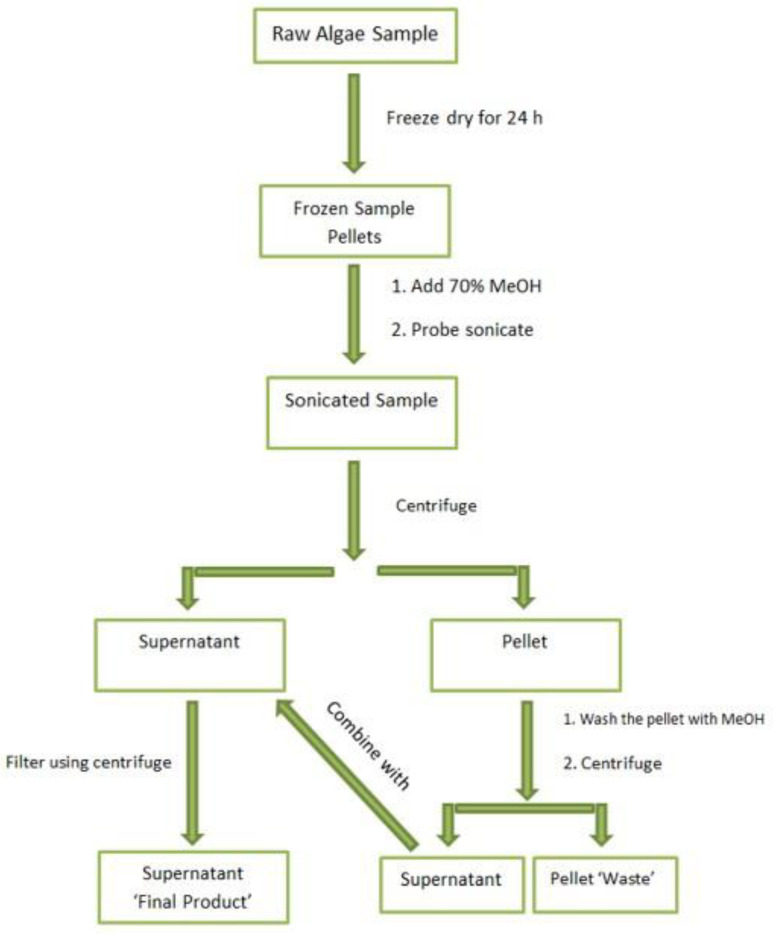
Schematic of MCs’ sample extraction workflow process.

Approximately 5 mg of dry algae was weighed and then probe-sonicated twice with 400 µL of 70% *v*/*v* LC-MS-grade methanol [21,79] for 1 min at 70% amplitude. The sample was then centrifuged at 3000× *g* and 4 °C for 15 min, whereupon the supernatant was then transferred to a 0.2 µm pore membrane filter tube. The remaining pellet was washed and subsequently extracted via centrifugation two more times with 400 µL of 70% *v*/*v* methanol. The supernatants were combined with the previous supernatant in the filter tube, which was then centrifuged for 30 min at 5000× *g*, resulting in the ‘Final Product’. Figure 7 provides a schematic representation of the MC extraction. 

#### 3.2.3. Standard and Sample Preparation and Derivatisation 

A standard curve range of 0.1–100 ng mL^−1^ of BMAA isomers (internal standard: 50 ng mL^−1^ D5-DAB) and 0.5–1000 ng mL^−1^ of MC standards (internal standard: 50 ng mL^−1^ nodularin) were prepared. The BMAA, AEG, 2,4-DAB, MC-RR, MC-YR, and MC-LR standards were combined and derivatised using an AccQ-Tag Ultra Derivatisation Kit in accordance with the manufacturer’s guidelines, with minor modifications. AccQ-Tag derivatisation in this method was carried out with a 10 µL BMAA standard/’Final Product’ sample, a 10 µL MC standard/’Final Product’ sample, 20 µL of derivatising reagent, and 60 µL of borate buffer, as opposed to 10:20:70 (sample:reagent:buffer). The BMAA, AEG, 2,4-DAB, and MC extracts of the cyanobacteria samples were combined with 500 ng mL^−1^ of nodularin prior to AccQ-Tag derivatisation. 

### 3.3. LC-MS/MS Analysis 

Analysis was performed on an Agilent 1290 series Infinity UPLC coupled with an Agilent 6490 Triple-Quadrupole LC-MS (Agilent Technologies—Mulgrave, Victoria Australia). All data analysis was performed using Agilent MassHunter Qualitative and Quantitative Analysis Software. The method optimisation, including source conditions, was performed on Agilent MassHunter Optimiser and Source Optimiser software. 

Chromatographic analysis was carried out on an Agilent 2.1 × 100 mm, 1.8 μm, reverse-phase Zorbax RRHD Eclipse Plus C18 column, with the column temperature set to 18 °C. Separation was achieved by gradient elution at 0.65 mL min^−1^, with the initial conditions set at 100% A (ultrapure water + 0.1% *v*/*v* formic acid (Sigma-Aldrich, Castle Hill, NSW, Australia)), increasing to 10% B (acetonitrile (Sigma-Aldrich, Castle Hill, NSW, Australia) + 0.1% *v*/*v* formic acid) over 6 min, then 33% B in the 7th minute, and subsequently increasing to 45% B at 10 min. At 10 min, the column was washed for 2 min at 100% B and equilibrated under the initial conditions for another 2 min. The injection volume of the samples was 5 μL, with each sample and standard injected in triplicate. 

Multiple reaction monitoring (MRM) transitions were established for each analyte and internal standard, with each compound having at least 3 MRM transitions, 1 quantifier ion transition, and 2 qualifier ion transitions. To ensure maximum sensitivity, acquisition segments were implemented, where BMAA, AEG, and 2,4-DAB were acquired between 3 and 7 min and the MCs between 7 and 10 min. Outside of these acquisition windows, the flow was diverted to waste. The collision energies accompanying the MRM transitions (see Table 4) were optimised using MassHunter Optimiser software. 

The electrospray ionisation (ESI) source settings were optimised for BMAA, AEG, and 2,4-DAB, as well as the MCs’ protonation. The ESI probe was supplied with nitrogen gas, and the method was run in positive ionisation mode, with the nebulising pressure of 20 psi and the iFunnel voltage at 110–200 V. The drying gas temperature was optimised and set to 210 °C, with gas flow of 14.0 L min^−1^. The sheath gas temperature was set to 400 °C at 11.0 L min^−1^. The capillary voltage was operating at 3000 V, and the nozzle voltage at 1500 V.

### 3.4. Method Validation Parameters 

The method was validated to ensure its adherence to the available WHO guidelines regarding exposure to the MCs. External calibration standards with ranges of 0.1 to 100 ng mL^−1^ for BMAA and its isomers and 0.1 to 1000 ng mL^−1^ for the MC standards were used. Each standard was injected in triplicate, with repeatability determined after 7 injections of the 10 ng mL^−1^ standard, and inter-day precision was determined based on a 7-day reproducibility test. The LOD was calculated based on the 0.1 ng mL^−1^ standard as 3.3× the signal-to-noise ratio (S/N), and the LOQ was calculated as 7× the S/N. Spike-recovery tests were conducted to assess the accuracy of the method in the sample matrix of interest, which involved the spiking of an algal sample with a 10 ng mL^−1^ spike and comparing the signal to a 10 ng mL^−1^ standard. Various other parameters were assessed during the method development, and they are discussed in Section 3.

### 3.5. Data Analysis

All data processing was carried out using Agilent MassHunter Software and Microsoft Excel. Cyanotoxin concentration (C) was determined against the calibration curve in ng mL^−1^ and then normalised to the dry weight (M) and reconstitution/wash volume of the sample (V), with respect to the dilution factor (*Di. F*) (Equation (1)).

(1) $$Normalised\;Mass\left(ng\;{mg}^{-1}\right)=\frac{C\left(ng\;{mL}^{-1}\right)\times V\left(mL\right)\times Di.\;F}{M(mg)}$$

## 4. Conclusions

We developed a multiclass LC-MS/MS method for the separation and quantification of BMAA, AEG, 2,4-DAB, MC-LR, MC-RR, and MC-YR in cyanobacteria in a single analytical run. The method was validated by assessing the linearity, accuracy, precision, and detection and quantification limits, and it was adequate for the quantitative determination of multiclass toxins in cyanobacterial samples. Since cyanobacteria have the potential to have a detrimental effect on human health and a negative impact on surrounding ecosystems, it is important that routine cyanotoxin analyses are performed. Combined cyanotoxin screening will not only provide greater efficiency in terms of time and cost but will also expand our understanding of the range of toxins produced by individual cyanobacterial species. 

## Figures and Tables

**Table 1:** Method validation linearity results and recovery rates for BMAA isomers and MCs.

Compound	Correlation Coefficient (r)	Linear Range (ng mL^−1^)	LOD (ng mL^−1^)	LOQ (ng mL^−1^)	% Recovery of a Spiked Sample
BMAA	0.9971	2–500	0.46	1.38	107.1
AEG	0.9964	2–500	0.43	1.29	93.4
2,4-DAB	0.9967	2–500	0.42	1.27	85.5
MC-RR	0.9982	0.5–500	0.13	0.40	94.6
MC-YR	0.9975	0.5–500	0.30	0.90	106.0
MC-LR	0.9971	0.5–500	0.28	0.85	109.3

**Table 2:** Method validation precision (%RSD) through intra- and inter-day (7-day) repeatability analysis, based on average peak area (*n* = 7) and retention time.

	Peak Area %RSD			Retention Time %RSD		
	Intra-Day	Inter-Day		Intra-Day	Inter-Day	
		Day 1	Day 2		Day 1	Day 2
BMAA	4.9	1.2	1.1	0.3	0	0.2
AEG	5.1	6.6	3.8	0.2	0	0.2
2,4-DAB	4.8	1.9	2.0	0.3	0.1	0.4
MC-RR	2.1	1.7	3.1	0.3	0	0
MC-YR	2.9	2.8	2.9	0.3	0.1	0
MC-LR	3.0	1.5	2.7	0.3	0.1	0

**Table 3:** Cyanotoxin detection and concentration (DW) of cyanobacterial samples to 3 significant figures (N.D. = not detected) (*n* = 3).

Compound	*Microcystis aeruginosa* DW (ng mg^−1^) ± SEM	Gunbower Scum DW (ng mg^−1^) ± SEM
BMAA	N.D.	N.D.
AEG	N.D.	N.D.
2,4-DAB	0.249 ± 0.062	N.D.
MC-RR	N.D.	135 ± 4.80
MC-YR	N.D.	N.D.
MC-LR	4830 ± 721	47.2 ± 19.3

**Table 4:** MRM transitions for all analytes using LC-QqQ-MS; * denotes quantifier ions.

Sample	Retention Time (min)	Precursor Ion (*m*/*z*)	Product Ions (*m*/*z*)	Collision Energy (eV)	Dwell Time (ms)
BMAA	5.28	459	119	30	50
			171	35	
			258 *	30	
			289	20	
AEG	5.53	459	119	30	50
			171	35	
			214 *	35	
			289	20	
2,4-DAB	6.01	459	119	30	50
			171	35	
			188 *	35	
			289	20	
D_5_-DAB	5.97	464	123	18	50
			145 *	30	
			171	20	
MC-RR	7.86	520	103	70	50
			127	50	
			135 *	30	
NOD	8.30	826	103	70	50
			135 *	60	
			227	50	
MC-YR	8.74	1046	127	80	50
			213	72	
		523	135 *	75	
MC-LR	8.88	996	112	68	50
			213	76	
		498	135 *	70	

## Data Availability

Data is contained within the manuscript.

## Sample Availability

Samples were depleted during the experiment.
